# MethylMasteR: A Comparison and Customization of Methylation-Based Copy Number Variation Calling Software in Cancers Harboring Large Scale Chromosomal Deletions

**DOI:** 10.3389/fbinf.2022.859828

**Published:** 2022-04-12

**Authors:** Michael P. Mariani, Jennifer A. Chen, Ze Zhang, Steven C. Pike, Lucas A. Salas

**Affiliations:** 1 Geisel School of Medicine, Department of Epidemiology, Dartmouth College, Hanover, NH, United States; 2 Geisel School of Medicine, Department of Biomedical Data Science, Dartmouth College, Hanover, NH, United States; 3 Guarini School of Graduate and Advanced Studies, Quantitative Biomedical Sciences, Dartmouth College, Hanover, NH, United States; 4 Guarini School of Graduate and Advanced Studies, Integrative Neuroscience at Dartmouth, Dartmouth College, Hanover, NH, United States

**Keywords:** methylmaster, copy number variation, DNA methylation, kidney cancer, clear cell renal cell carcinoma, epigenetics, genomics, multiomics

## Abstract

DNA methylation-based copy number variation (CNV) calling software offers the advantages of providing both genetic (copy-number) and epigenetic (methylation) state information from a single genomic library. This method is advantageous when looking at large-scale chromosomal rearrangements such as the loss of the short arm of chromosome 3 (3p) in renal cell carcinoma and the codeletion of the short arm of chromosome 1 and the long arm of chromosome 19 (1p/19q) commonly seen in histologically defined oligodendrogliomas. Herein, we present MethylMasteR: a software framework that facilitates the standardization and customization of methylation-based CNV calling algorithms in a single R package deployed using the Docker software framework. This framework allows for the easy comparison of the performance and the large-scale CNV event identification capability of four common methylation-based CNV callers. Additionally, we incorporated our custom routine, which was among the best performing routines. We employed the Affymetrix 6.0 SNP Chip results as a gold standard against which to compare large-scale event recall. As there are disparities within the software calling algorithms themselves, no single software is likely to perform best for all samples and all combinations of parameters. The employment of a standardized software framework via creating a Docker image and its subsequent deployment as a Docker container allows researchers to efficiently compare algorithms and lends itself to the development of modified workflows such as the custom workflow we have developed. Researchers can now use the MethylMasteR software for their methylation-based CNV calling needs and follow our software deployment framework. We will continue to refine our methodology in the future with a specific focus on identifying large-scale chromosomal rearrangements in cancer methylation data.

## INTRODUCTION

CNV calling from DNA methylation data remains an attractive prospect as both genetic (copy-number) and epigenetic (methylation) data can be obtained from the same experimental results. There are, thanks to Illumina, a pedigree of methylation analysis platforms available to the genomicist. Beginning with the Illumina Infinium HumanMethylation27 or “27k” BeadArray platform, followed by the widely used Illumina Infinium HumanMethylation450 or “450k” BeadChip (Bibikova et al., 2011), and finally, the Illumina Infinium MethylationEPIC or “EPIC” platform, released in 2015, which sports over 850,000 methylation probes (Sandoval et al., 2011; Triche et al., 2013; Moran et al., 2016). With each generation, the researcher is availed of more comprehensive genome coverage from the added probe density (Kilaru et al., 2020). DNA methylation arrays measure bisulfite converted unmethylated cytosines as thymines. In contrast, methylated cytosines are protected from the bisulfite conversion and remain as cytosines (Aryee et al., 2014). The CNV status can then be inferred from the relative ratio of converted thymines to remaining cytosines (red and green channel intensities) (Feber et al., 2014).

SNP array-based comparative genome hybridization retains the greatest resolution and is still used as the gold standard in several public databases (Koike et al., 2011; Kilaru et al., 2020). Some time after this technology was developed, researchers later sought to identify CNVs from DNA methylation data, the need to process this data was first answered with software such as DNAcopy (Seshan and Olshen, 2021) and Minfi (Aryee et al.). Shortly thereafter, Feber et al. built off these initial software with their release of ChAMP (Morris et al., 2014; Tian et al., 2017). After ChAMP followed the CopyNumber450kCancer (Marzouka et al., 2016) and cnAnalysis450k (Knoll et al., 2017) routines which were specifically aimed at analyzing the 450k platform data.

These arrays have been validated in a variety of studies since their inception, distribution, and utilization for cancer studies, among others: e.g., 450k with colorectal cancer (Sandoval et al., 2011), again the 450k BeadChip for acute lymphoblastic leukemia (Nordlund et al., 2013), or combined with other copy-number and transcriptomic data for multi-omics studies in glioblastoma (Sturm et al., 2012). Researchers have pioneered improvement at each stage of technology development in their application, including software and wet-lab improvements that have provided the genomics community with additional accuracy and inferential capabilities—such as normalization improvements to signals generated from the 450k platform (Fortin et al., 2014).

Feber et al. described several technical challenges in the processing and analysis of DNA methylation array data. However, they also identified how CNV calling from methylation data could potentially identify translocations and inversions while the earlier genotyping arrays cannot. Additionally while, the genotyping arrays have a very high resolution, methylation-based CNV callers have an easier time identifying CNVs in genes relative to intergenic regions (Feuk et al., 2006; Feber et al., 2014; Kilaru et al., 2020).

450k platform-specific software like CopyNumber450kCancer and cnAnalysis450k incorporated computational and statistical methodology in novel ways to address these unique technical challenges. For example, the latter borrowed normalization routines from other analytical routines such as the dasen family of preprocessing methods (wateRmelon) (Pidsley et al., 2013), and ssNoob, Quantile, Funnorm, and SWAN—all part of minfi (Aryee et al., 2014). Anotherkey finding was that the normalization distributions were skewed after comparing these procedures (Knoll et al., 2017). This skewing meant differing gain/loss cutoffs would have to be applied depending on the normalization method. During the DNA methylation array processing, the copy-number inference is based on the red (unmethylated signal) and green (methylated signal) intensity channels. Technical variations in these two signal channels need to be accounted for as the relative differences between these signals allow the CNV state to be inferred. Thus, changes in intensity normalization may affect CNV calling, but not necessarily the methylation calling (Knoll et al., 2017). To alleviate both the baseline offset and distribution skew issues, the cnAnalysis450k authors chose a normalization procedure that implements a z-transformation that scales data sets relative to one another and is purported to alleviate the above issues (Knoll et al., 2017).

Even so, analytical challenges persisted, as regions that harbored genomic deletions were still producing false-positive artefactual spurious signals (Zhou et al., 2018). Zhou et al. attempted to resolve this issue by developing a statistical approach to masking deleted or “hyperpolymorphic” genomic regions (Zhou et al., 2018). Their software, SeSAMe, implemented a technique called pOOBAH or *p*-value with out-of-band (OOB) array hybridization. They report reducing the number of false-positive epigenetic silencing regions reported. The focus was on the tumor suppressor genes CDKN2A and RB1, often deleted in tumors (somatic deletions). The authors also claimed that their method decreased technical variation and retained biological variation across 450k and EPIC platform samples. Finally, they claim that the SeSAMe package is suitable for efficiently analyzing thousands of samples, such as those from The Cancer Genome Atlas (TCGA), which we analyze in this paper (Triche et al., 2013; Zhou et al., 2018).

Most recently, in 2019, the authors of Epicopy (Cho et al., 2019) sought to use existing tools to streamline the analysis and improve accuracy for TCGA datasets. They used functional normalization (Fortin et al., 2014) and like the other methods mentioned above, built off of the methodology of, or incorporated, DNAcopy to perform methylation signal mean- centering, circular binary segmentation, and copy number estimation (Olshen et al., 2004; Feber et al., 2014; Cho et al., 2019). They applied the GISTIC 2.0 (Mermel et al., 2011) software to get copy numbers for individual genes and samples as well as to find “focal” and “arm-level” events in each tumor type (Cho et al., 2019). Our software, MethylMasteR, integrates the four different routines described above: SeSAMe, our own version of cnAnalysis450k (Called “HM450” by us), ChAMP, and Epicopy. Other CNV calling methods that have been developed along the way—such as conumee (Hovestadt and Zapatka, 2017), as well as newer methods that continue to be developed to help address the ongoing analytical challenges discussed above. See Kilaru et al., 2020 for a review (Kilaru et al., 2020). More recently, methodology is also being developed to explore methylation and copy number variation within the context of single cell Multiomics as well (Hou et al., 2016; Bian et al., 2018).

While CNV calling can be performed from DNA methylation arrays, the gold standard remains high-density SNP arrays (Koike et al., 2011; Feber et al., 2014; Cho et al., 2019). It is desirable to perform genetic (copy number variation) and epigenetic (methylation) analyses at the same time via methylation data (rather than using separate SNP Chips to identify CNV’s to reduce costs and materials used (Feber et al., 2014), but researchers need to be sure that the methods available to call CNVs from methylation data are accurate and performant under various conditions. Studies need to be performed to assess said performance and accuracy of the pipelines available to accomplish this task.

Herein, we present MethylMasteR, a software that provides a stable and user-friendly platform for running DNA methylation- based CNV calling algorithms. MethylMasteR allows for the comparison between the performance (total runtime and peak memory usage), and the ability to identify large-scale CNVs (recall) in cancer samples, across four popular algorithms that call CNVs from methylation data. We also introduce our custom routine incorporating SeSAMe and parts of the CopyNumber450kCancer CNV calling routines. The MethylMasteR software package combines the DNA methylation CNV callers using a common Sample Sheet and raw IDAT input files. Individual algorithms are then run, and CNV segments are transformed into a common data. frame format, which facilitates visualizations and comparison across algorithms. Finally, and perhaps most importantly, we have implemented our software within the Docker software architecture (Merkel, 2014) which allows us, the developer to control software dependency harmonization and versioning without hassle to the end-user.

## METHODS

### Sample Selection

A total of 31 kidney cancer samples (KIRC) from the Firehose database on GDC’s TCGA were selected based on the status of their VHL-coding region on the short arm of chromosome 3 (3p). All 31 samples were selected to contain deep 3p deletions associated with clear cell renal cell carcinomas. The corresponding Illumina 450k BeadChip IDAT files were downloaded from TCGA. A further 50 low-grade glioma (LGG), subtype oligodendroglioma, samples were also downloaded from the Firehose database. Twenty-five samples are histologically defined oligodendrogliomas and contained codeletion of the short arm of chromosome 1 and the long arm of chromosome 19 (1p/19q), and the other twenty-five samples were astrocyte-like oligodendrogliomas that were copy-number neutral in these regions (Li et al., 2016). The Affymetrix SNP Array 6.0 (“gold standard”) copy number segmentation data corresponding to the above data was also downloaded from TCGA.

All testing was performed on a Dell Precision 5,820 Tower X-series workstation running Windows 10 Pro 64bit and R 4.1.2. 128 GB of useable RAM and an Intel(R) Core(TM) i9-10900X CPU x64 processor @ 3.70 GHz. All analyses were performed only using a single core as some packages set multiple cores for the analysis; we wanted to ensure equal computational resources were allocated for each routine.

### Copy Number Variation Calling Methods

We ran four main workflows. All workflows use the same general formula to calculate the copy number state from the original methylation signal intensities that we find in the raw IDAT files: a log ratio is first calculated between the methylation signal of a test set (I_T_) over that ofa reference set (I_R_). This could be a ratio of tumor sample intensities over normal sample intensities or over a reference intensity set such as the Epic.5. normal default samples used by SeSAMe, or the median signal intensity used by default in Epicopy. Thus $LRR=log_{2}\left(\frac{I_{T}}{I_{R}}\right)$. Then a threshold is applied to the LRR values to determine the copy number state of a particular region. We chose a more liberal threshold of −0.2 and 0.2 than what is often seen in literature (Feber et al., 2014) for copy number losses and gains respectively. Regions with LRRs within this range were consider neutral.

The first workflow that we ran is the Sesame workflow. SeSAMe and the sesameData R package were used to load in the “Epic.5. normal” reference (prostate-derived cell lines and consequently male-only samples) and the tumor. IDAT files from TCGA. The openSesame () function was used to read in the files, perform background correction using the normal-out-of-band (noob) algorithm (Fortin et al., 2014), a nonlinear dye bias correction step to account for differences in the red and green fluorescent dyes used to measure methylation (Zhou et al., 2018), and pOOBAH masking to identify and correct for aberrant methylation signal stemming from hybridization failure (Zhou et al., 2018) and output data as SeSAMe signal sets. Segmentation analysis was then performed with SeSAMe using the “Epic.5. normal” reference data set.

The MethylMasteR “HM450” workflow is based on the cnAnalysis450k routine. With this routine, SeSAMe was again used to read in and preprocess the control Epic.5. normal and tumor samples from IDAT files as described above. The preprocessed signal sets were then converted to an RGChannelSet object and extracted into a GenomicRatioSet object using the Minfi preprocessRaw() and getCN() functions for both the control and treatment samples (Aryee et al., 2014). The data in this object was then z-transformed for both the tumor and control data, and the median intensity values of all control samples were also taken. The CNV segments were then calculated from the above-transformed data and the median control intensity using cnAnalysis450k functionality. The final CNV calls were plotted as heatmaps following the style of CNAclinic (Chandrananda, 2017) and output as comma- separated-values files (CSVs).

ChAMP functionality was adjusted slightly and applied to the 31 KIRC deep 3p deletion samples. The function was modified to accept a modified Illumina-style sample sheet—the MethylMasteR Style sample sheet and the sample sheet path can be specified independently from the IDAT files path. The entire ChAMP routine was run with the modified champ. process () functionality, we disabled components that were not strictly necessary for CNV calling such as differential methylation region detection, as it would increase the overall run time, and possibly the peak memory usage as well. Again, heatmaps and CSVs for the CNVs were generated as outlined above.

For the Epicopy routine, the authors were unable to get the normal data object to load with a “magic number” error, suggesting that it may need to be regenerated for the newest version of Epicopy. In place of the built-in “thyroid,” “breast,” and “lung” normal (TCGA-derived normal-adjacent samples), the median was specified as the internal control to use for these samples. Heatmaps and CSVs were again generated. Again, as with ChAMP, some of the Epicopy (and Minfi) internal functionality was modified to use the MethylMasteR sample sheet and workflow.

Our custom routine mostly follows the SeSAMe methodology, but we added additional peak correction functionality to correct baseline offsets in the segmentation calls. This was done by employing the AutoCorrectPeak () functionality from CopyNumber450kCancer, which is meant to adjust the Log R Ratios (LRRs) baseline and improve the accuracy of CNV segment calls (Marzouka et al., 2016).

In each case, the final segmentation state was calculated by setting a segment mean LRR threshold to ≤ −0.2 and ≥0.2 to copy number losses and gains, respectively, for visualization. Any segment with a mean between these two values was considered neutral. The same threshold was used across all algorithms to keep the comparisons as equal as possible.

Finally, the gold standard 31 KIRC, and 50 LGG, samples from the firehose legacy portal SNP CNV calls from the Affymetrix SNP Array 6.0, were loaded into MethylMasteR using a custom routine. Heatmaps and CSVs were again generated against which the CNVs could be compared from the above four routines.

### Comparison

The peakRAM() function from the peakRAM R package (Quinn, 2017) was used in R to identify peak memory usage during each routine, and the base R Sys. time () function was used to calculate the total time elapsed.

For comparison of renal cell carcinoma CNV segments identified across routines, we chose to measure the recall of each routine against a “gold standard” reference data set: the hg19 SNP6 CNV segment data corresponding to our 31 KIRC firehose legacy test samples. The countOverlaps () function from the GenomicRanges R package (Lawrence et al., 2013) was used to calculate overlaps between CNV segments identified by the MethylMasteR routines and the gold standard segments (both tumor and normal adjacent) from the Affymetrix SNP6 chips. Here the recall was calculated as the percent of CNV reference segments correctly identified, $R=\frac{\left(TN+TP\right)}{TR}=\frac{ON+(OT-ONT)}{N\cup T}$. is calculated as the number of normal reference (N) (SNP6 gold standard) CNV segments that overlapped at least one routine CNV call by one or more bases. Overlaps tumor (OT) is calculated the same as ON but for the tumor reference CNV segments (T). Because true negatives were prioritized over true positives, segments that overlapped between the normal standard and tumor standard (ONT) were subtracted from OT to get the true positive (TP) number of reference segments. The total number of reference segments (TR) is the total number of segments that comprises the union of the normal reference segments (N) and tumor reference segments (T) calculated with the GenomicRanges union () function. ONT was calculated using the seqsetvis R package (Boyd, 2021).

For the oligodendroglioma samples, the recall was calculated, and comparisons were performed similarly to the above with a couple of adjustments. Because there was no tissue adjacent Affymetrix SNP6 data, we used the 25 histologically determined oligodendroglioma samples, harboring the 1p/19q codeletions as the test group and the 25 astrocyte-like oligodendroglioma samples that were neutral for these deletions as the normal group. To determine which samples contained the deletions or were neutral, the mean LRR values were retrieved from cBioPortal. Any CNV with mean intensity ≤ −0.2 was labeled a deletion (copy number = 1), ≥0.2 as an amplification (copy number = 3), and in-between values were labelled as neutral (copy number = 2). If a sample harbored no CNVs with a copy number value = 1 and met a detection threshold to ensure a large-scale event (total marks for that CNV >10,000) in both 1p and 19q, it was classified as a tumor (T) reference sample. If instead, the state was equal to 2 with the other parameters being the same, it was classified as a normal (N) reference sample.

## RESULTS

**FIGURE 1 |:**
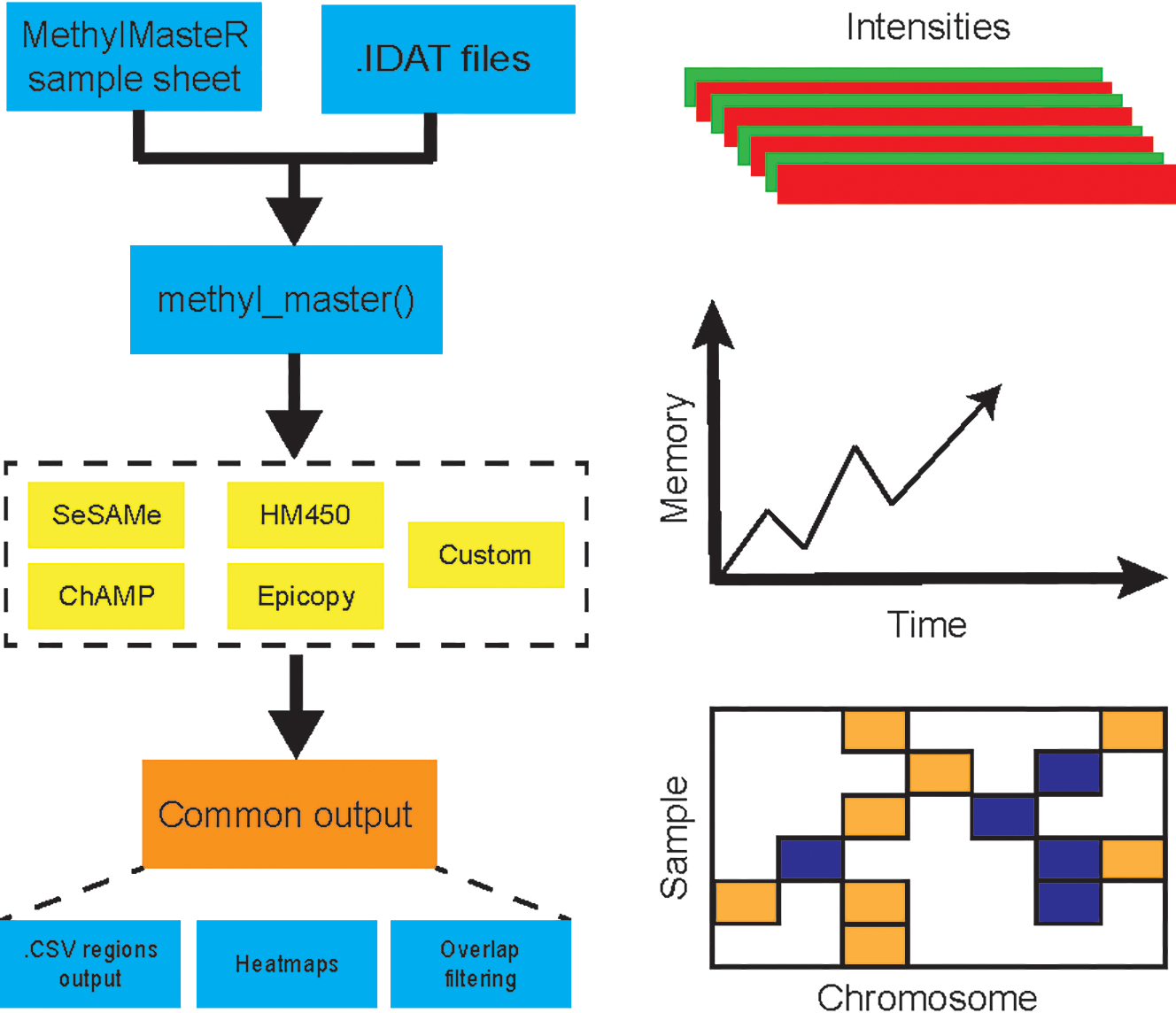
MethylMasteR workflow overview. The MethylMasteR software package combines common methylation CNV callers using a common Sample Sheet and raw IDAT files and is implemented as a Docker image which can be run as a Docker container on any operating system that has Docker installed. Individual algorithms are then run, and CNV segments are formatted into a common data. frame format, which facilitates visualizations and comparison across algorithms. In addition to the generation of CNV heatmaps and tables, time and memory are also recorded for comparison across algorithms.

**FIGURE 2 |:**
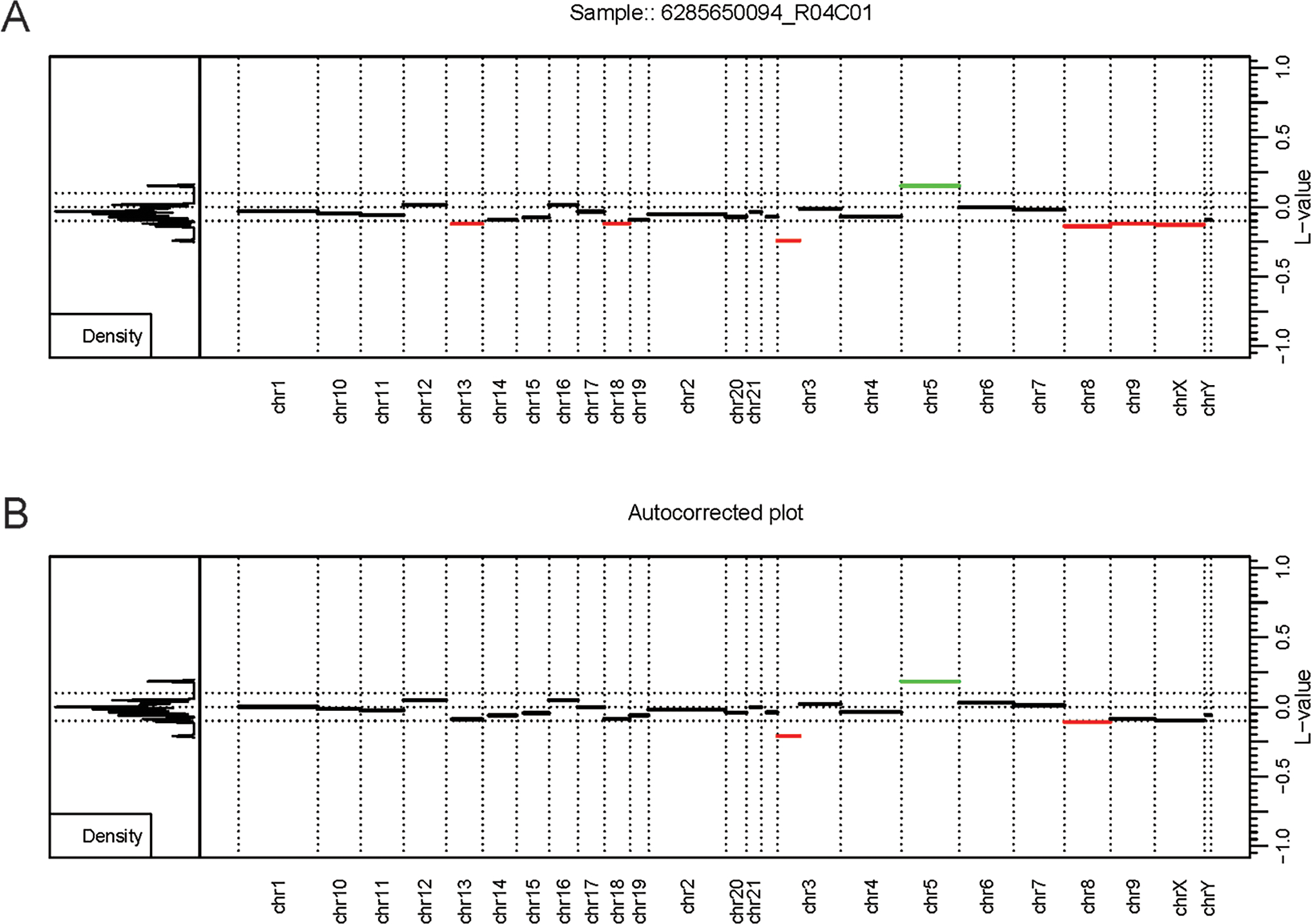
Autocorrection as part of the custom workflow. The MethylMasteR software framework enables the easy deployment of custom workflows such as results from the SeSAMe and CnAnalysis450kCancer workflows above. (A) An uncorrected CopyNumber450kCancer-style plot. (B) The corrected CNV segmentation values after processing with SeSAMe segmentation values with the AutoCorrectPeak () function from the CopyNumber450kCancer R package. Abbreviations: L-value - the LRR value or log2 ratio of tumor methyla.

**FIGURE 3 |:**
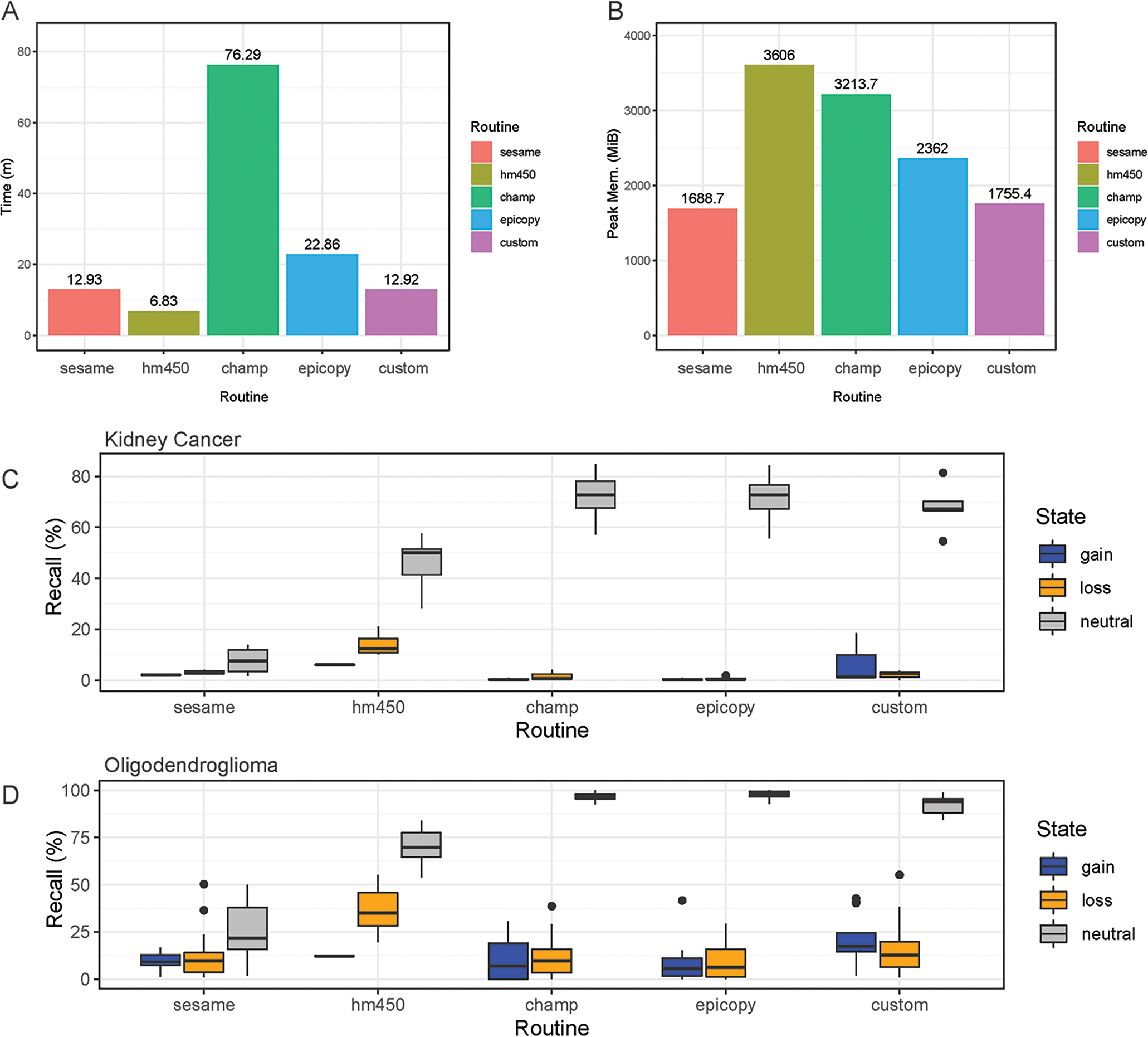
Comparisons of CNV calling routines. Results are shown for the four common software routines incorporated into the MethylMasteR framework and the MethylMasteR custom analysis routine. (A-B). ChAMP required an extended runtime and peak memory for successful completion, while HM450 was fast but memory intensive. SeSAMe and the custom routine were optimal for speed and memory usage, with Epicopy a close second. (C-D). Overlaps with the Affymetrix SNP6 legacy data as the gold standard showed that Epicopy was the least accurate overall, with ChAMP having slightly better recall for identification of losses in kidney cancer but better recall for gains in oligodendroglioma. HM450 and our custom routine were the most accurate overall, and HM450 required less run time but used the highest amount of peak RAM of all the routines (except the copy number neutral astrocyte-like oligodendroglioma runtime results—see Supplementary Table S1.1). SeSAMe was not as accurate in identifying losses as our custom routine and had similar run time performance. This routine was also on par with ChAMP and Epicopy in terms of recall for the oligodendroglioma samples. In general, similar recall results to those obtained for the renal cell carcinoma samples in Figure 3C can be seen for the oligodendroglioma samples as well in Figure 3D.

Overall, the analysis provided insightful information about individual routines performance within the standardized framework provided by our software and outlined in Figure 1. Our framework also facilitates the implementation of custom functionality, such as peak correction, exemplified in Figure 2. The baseline-corrected segments (Figure 2B) have fewer false positives at the −0.2,0.2 LRR level vs the uncorrected segments (Figure 2A). The MethylMasteR program completed each subroutine successfully, and the results of the individual routines relative to one another can be seen in Figure 3.

ChAMP took over 1 h to complete the renal cell carcinoma data with the 31 test samples. Many features were disabled, including batch correction, differential methylation region calling, and gene set enrichment analysis. In addition, ChAMP was pretty memory intensive, requiring over 2 Gigabytes of RAM at its peak. The HM450 routine required the most RAM at its peak but completed the fastest by far at well under 10 min. Epicopy took the middle ground regarding time and memory usage, while SeSAMe and our closely related Overlaps normal (ON) is the true negative number (TN) and custom routine had the best combined time and memory performance (Figures 2A,B). The above performance results can also be found in Supplementary Table S1.1. These patterns were observed when running the routines on the oligodendroglioma samples as well with the exception that ChAMP, Epicopy, and our custom routine required more memory than expected for the astrocyte-like (copy number neutral) oligodendroglioma samples compared to the other two categories: renal cell carcinoma and histologically determined oligodendroglioma (codeletion) samples (Supplementary Table S1.1).

We can see that Epicopy, and ChAMP performed the worst in terms of recall for copy number gains and losses for the kidney cancer samples but had improved performance, most notably with ChAMP in terms of identifying gains, in the oligodendroglioma samples. Both routines identified many neutral regions in both tumor types (Figures 3C,D). SeSAMe was able to recognize the gains and losses very well but had some trouble identifying neutral regions against the gold standard SNP6 Chip CNV calls. The HM450 routine and our custom pipeline performed the best in terms of recall, but HM450 used a high level of total peak RAM. In contrast, both SeSAMe and our custom routine (which builds on SeSAMe results) were slower than the HM450 routine (Figures 3A–D). A comparison of the final CNV states of our custom routine to the SNP6 standard (both tumor and normal) can be seen in Supplementary Figure S1. All recall results are also contained in Supplementary Table S1.2 (renal cell carcinoma) and Supplementary Table S1.3 (low- grade glioma: oligodendroglioma).

## DISCUSSION

Overall, our custom routine, SeSAMe, and HM450 performed the best in execution time, memory, and overall recall. Our custom workflow, which builds off SeSAMe, had an advantage in identifying gains and copy number neutral regions over SeSAMe alone. HM450 was best overall at identifying losses - an important finding when considering that copy number losses in the short arm of chromosome 3 are essential drivers in many kidney cancers. In addition, the large-scale loss of 1p and 19q essential for the molecular diagnosis of classical oligodendrogliomas was most easily identified using HM450 as well, which thus demonstrated decent recall and run time but required the most peak RAM usage of any of the routines. This is an essential factor to consider when analyzing many samples. ChAMP and Epicopy appeared to perform the worst overall for the kidney cancer results but performed better for the oligodendroglioma samples. The many dependencies required for both of these routines may lead to a greater number of parameters that need to be fine-tuned for individual tumor and may be responsible for the variability in recall across tumor types. ChAMP held a slight edge in recall relative to Epicopy but required a significantly longer runtime and more peak RAM usage. There was also some divergence in expected memory usage in the copy-number neutral oligodendroglioma samples relative to the other two categories in ChAMP, Epicopy, and our custom routine as well. This demonstrates that while general patterns were observed, one must be cautious when analyzing and interpreting memory requirements for complex software that builds upon many dependencies, which are not all necessarily designed with memory optimization in hand. In addition, many of the available ChAMP features that were not essential for CNV segmentation calling were not run; thus, in many user cases, the runtime for ChAMP would be even longer. It should be noted that ChAMP offers parallel processing support that would speed up computation time, but it is not clear that this feature is supported or desirable on all operating systems due to the high RAM consumption during the serial processing.

Overall, it is crucial to note that the recall for calling CNV’s using methylation data is low relative to the gold standard SNP6 results. Yet, to tumor biologists, large-scale chromosomal aberrations are of great biological interest, and we have demonstrated that some routines perform better than others in this regard. Indeed, the identification of such events is mentioned as an advantage to CNV calling with methylation data in some cases (Feber et al., 2014). At the same time, the resolution of the SNP-based CNV calling methods ensures that they will remain the gold standard for now. As we have reproduced herein, CNV calling with DNA methylation data can identify large-scale CNV events and has the advantage of providing DNA methylation information in parallel. Thus, the researcher will have to decide which cases are best suited for CNV calling with methylation data and which, or how many, analysis routines to run (Kilaru et al., 2020).

The overall formatting of the software in a standardized manner allowed us to quickly implement our custom routine involving the primary SeSAMe analysis pipeline with a modified version of the AutoCorrectPeak () function originally from the CopyNumber450kCancer pipeline. The organization of the MethylMasteR framework allowed us to push the overall gain and loss recall almost to the level of the HM450 routine, which was the best at identifying large-scale copy number losses such as the chromosome 3p deletion commonly seen in renal carcinoma. Similarly, by modifying the Ilumina-style sample sheet that is used by minfi, ChAMP, and Epicopy, to our MethylMasteR sample sheet, we have incorporated a single input sample file that can easily be used across all the routines in our software. This feature added to our ability to streamline comparisons across the various algorithms and parameters; similarly, we can create a more efficient software platform to facilitate downstream analyses across the multiple routines by using the same internal data formats.

The software also has additional features that allow the user to customize analyses. For instance, specifying a normal control within the sample sheet (instead of using the internal references) and the gathering of a final set of consensus regions (also output in. CSV format) using a modified version of population_ranges () function from CNVRanger (da Silva et al., 2020) are two such features. The population_ranges () function was called with default parameters: density = 0.1, rho = 0.5, and est. recur = TRUE. Another example of analysis customization is that MethylMasteR allows the user to change the LRR signal threshold parameter that is used identify copy number states. In addition to thresholds, the R equation: seg.state round 2^seg.means^*2 can be used by setting this parameter to “NULL” in R. Such intelligent parameter deployment is another example of the analytical versatility provided by MethylMasteR. Finally, the standardized visualization of CNV states across routines via heatmaps is useful and novel and allows for the fast and easy interpretation of data.

Finally, and perhaps most importantly, we decided to build our software into the Docker architecture, creating an image that can be downloaded and run as a container on any operating system that has Docker installed. Because Docker is now available for so many operating systems, this provides an excellent, novel way to regulate software version control and compatibility on our end as developers and release the final product as a standalone executable for the user to run within a Docker environment. This approach reduces the added difficulty that many users experience when downloading, installing, or integrating various genomics software - all of which may not be actively maintained.

Overall, we have demonstrated how multiple DNA methylation software can be combined in a comprehensive yet efficient framework for accurately calling large-scale CNVs from raw DNA methylation signals and comparing their performance across algorithms. In the future, we plan to add additional functionality to the custom routine to improve MethylMasteR’s recall and overall accuracy further. We also will continue to refine our Docker-based approach, allowing users ease of use, and developers maximum control over, the development of genomics software requiring numerous versioned dependencies. Such a framework will enable researchers to explore the genetic and epigenetic cancer biology that can be gleaned from DNA methylation data with greater facility and accuracy.

## Supplementary Material

Supplementary Tables S1.1-3

Supplementary Figure S1

## Data Availability

Publicly available datasets were analyzed in this study. This data can be found here: The results shown here are in whole or part based upon data generated by the TCGA Research Network: https://www.cancer.gov/tcga. Data from TCGA are available via the Genomic Data Commons (GDC).
