# Supplementary material for: MethylMasteR: A Comparison and Customization of Methylation-Based Copy Number Variation Calling Software in Cancers Harboring Large Scale Chromosomal Deletions
Source: Front Bioinform. 2022 Apr 12;2:859828. doi: 10.3389/fbinf.2022.859828 (PMC9098103; doi:10.3389/fbinf.2022.859828)
Supplement: Supplementary file 2 [file Image1.pdf]

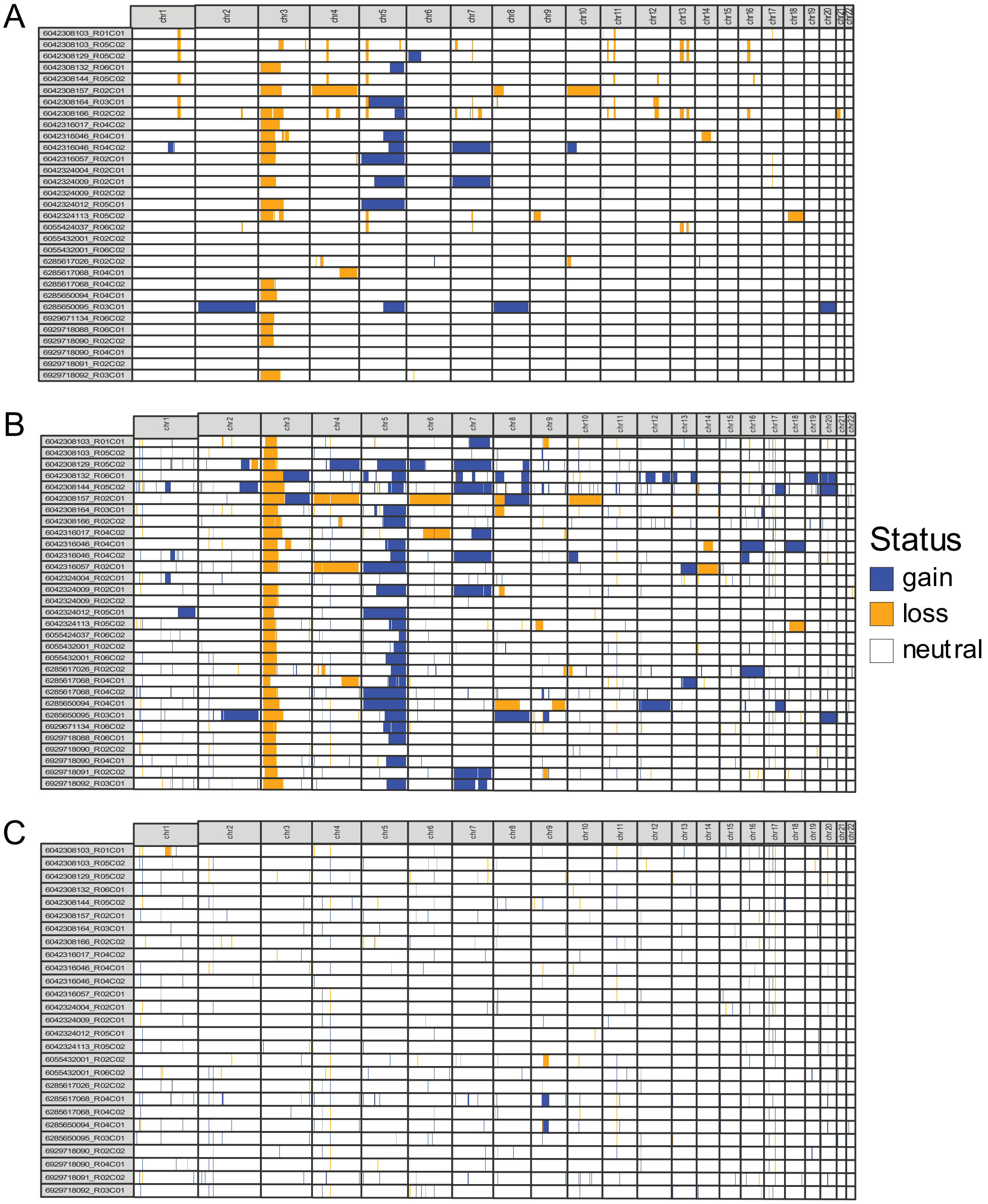

Supplementary Figure S1. MethylMasteR and gold standard heatmaps of CNV calls. Plots were generated with ggplot2 and meant to be in the style of a CNAclinic (Chandrananda 2017) heatmap that the authors of this paper like for visual comparison across chromosomes. **A**. our custom workflow captured many of the large-scale CNV events such as the losses and gains on chromosomes 3 and 5 respectively. **B-C**. Gold standard CNV states calculated from Affymetrix SNP6 TCGA firehose legacy datasets corresponding to the 31 clear cell renal cell carcinoma (KIRC) TCGA samples analyzed in this paper. Tumor (**B**) and normal-adjacent (**C**) are both shown.
