# Supplementary material for: MethylMasteR: A Comparison and Customization of Methylation-Based Copy Number Variation Calling Software in Cancers Harboring Large Scale Chromosomal Deletions
Source: Front Bioinform. Author manuscript; Available in PMC 2022 May 12. (PMC9098103; doi:10.3389/fbinf.2022.859828)
Supplement: Supplementary file 1 [file NIHMS1792751-supplement-Supplementary_Figure_S1.docx]

**Supplemental Figure 1. MethylMasteR and gold standard heatmaps of CNV calls**. Plots were generated with ggplot2 and meant to be in the style of a CNAclinic (Chandrananda 2017) heatmap that the authors of this paper like for visual comparison across chromosomes. **A**. our custom workflow captured many of the large-scale CNV events such as the losses and gains on chromosomes 3 and 5 respectively. **B-C**. Gold standard CNV states calculated from Affymetrix SNP6 TCGA firehose legacy datasets corresponding to the 31 clear cell renal cell carcinoma (KIRC) TCGA samples analyzed in this paper. Tumor (**B**) and normal-adjacent (**C**) are both shown.
